# Risk factors for recurrent injurious falls that require hospitalization for older adults with dementia: a population based study

**DOI:** 10.1186/s12883-016-0711-3

**Published:** 2016-09-29

**Authors:** Lynn B. Meuleners, Michelle L. Fraser, Max K. Bulsara, Kyle Chow, Jonathon Q. Ng

**Affiliations:** 1 Curtin-Monash Accident Research Centre (C-MARC), Curtin University, GPO Box U1987, Perth, 6845 WA Australia; 2 Eye & Vision Epidemiology Research Group (EVER), Perth, WA Australia; 3 Institute for Health Research, The University of Notre Dame, Fremantle, 6959 WA Australia; 4 Centre for Health Services Research, School Of Population Health, The University of Western Australia, 35 Stirling Highway, Crawley, 6009 WA Australia

**Keywords:** Dementia, Recurrent falls, Hospitalization, Population-based study

## Abstract

**Background:**

Older adults with dementia are at an increased risk of falls, however, little is known about risk factors for recurrent injurious falls (a subsequent fall after the first fall has occurred) among this group. This study aimed to identify risk factors for recurrent injurious falls requiring hospitalization among adults aged 60+ years with dementia.

**Methods:**

This retrospective, whole-population cohort study was conducted using the Western Australian Hospital Morbidity Data System and Western Australian Death Registrations from 2001 to 2013. Survival analysis using a stratified conditional Cox model (type 1) was undertaken to identify risk factors for recurrent injurious falls requiring hospitalization.

**Results:**

There were 32,519 participants with an index hospital admission with dementia during the study period. Over 27 % (*n* = 8970) of the cohort experienced a total of 11,073 injurious falls requiring hospitalization during follow up with 7297 individuals experiencing a single fall, 1330 experiencing two falls and 343 experiencing three or more falls. The median follow-up time for each individual was 2.49 years. Females were at a significantly increased risk of 7 % for recurrent injurious falls resulting in hospitalization (adjusted hazard ratio 1.07, 95 % CI 1.01–1.12), compared to males. Increasing age, living in rural areas, and having an injurious fall in the year prior to the index hospital admission with dementia also increased the risk of recurrent injurious falls resulting in hospitalization.

**Conclusions:**

Screening those with dementia for injurious falls history could help to identify those most at risk of recurrent injurious falls. Improvement of heath care and falls prevention services for those with dementia who live in rural areas may also reduce recurrent injurious falls.

## Background

In 2012, dementia was the third leading cause of death in Australia, accounting for 10,369 deaths [1]. The prevalence of dementia is increasing and by 2020 there will be an estimated 48 million people worldwide with dementia [2]. Dementia is not a specific disease but a clinical syndrome that comprises multiple diseases characterized by progressive deterioration in cognitive ability and a gradual, steady decline in memory, language, problem solving, judgment, and decision making [3].

As dementia progresses it can result in a person being more unsteady, prone to falls and wandering, confused, immunosuppressed, unable to care for themselves, forgetful and unaware of their surrounding environment [4]. This results in individuals with dementia being at increased risk of injuries, particularly falls, motor vehicle crashes, accidental poisoning, and burns [4–6].

Falls affect between 60 and 80 % of older adults with cognitive impairment [7, 8]. Injuries from a fall can represent a pivotal event for older people, resulting in loss of confidence, social isolation, decreased quality of life, declining physical health, institutionalization and death [9, 10].

Current evidence also suggests that those with dementia have two to eight times more falls than older individuals with no dementia or cognitive impairment [7, 8, 11]. This increased risk has been documented in a range of settings, including the community [7], long-term care settings/ nursing homes [11] and hospitals [8]. Previous research also found that the occurrence of a fall increased the risk of a subsequent fall [7, 11].

Prospective longitudinal studies examining risk factors associated with multiple falls in those with dementia are few and usually involved short follow-up periods [12, 13]. The frequency and severity of falls can vary widely and longer-term follow-up can highlight enormous intra- and inter-individual variability. Previous studies have also been limited by small sample sizes, recall bias, and the problems associated with self-reported measures [7, 12, 13].

The current study uses whole-population data to identify risk factors for recurrent injurious falls (a subsequent fall after the first fall has occurred) that resulted in hospitalization for older adults with dementia. It is anticipated that the results of this study may provide evidence for better targeting of interventions to reduce injury and impact on the health care system resulting from recurrent injurious falls among older people with dementia.

## Methods

### Study design

A retrospective whole-population cohort study was undertaken to determine risk factors for recurrent injurious falls that required hospital admission among adults aged 60+ years with dementia from 2001 to 2013.

### Data sources

De-identified data was obtained through the Western Australian Data Linkage System (WADLS). The WADLS is a validated, population-based, data linkage system that creates links among state health-related data sets [14]. It is one of only seven such record linkage systems in the world and allows retrospective studies to be conducted many years after exposure and eliminates the burden on respondents and reliance on self-reports. Using the WADLS also overcomes limitations due to small sample size, loss to follow-up and accurate ascertainment of exposures and outcome measures [14]. The largest component of the WADLS is the Hospital Morbidity Data System (HMDS) which contains all inpatient discharge summaries from all private or public WA hospitals since 1970. By law, all WA deaths are recorded in the WA Death Registrations.

### Case ascertainment

Cases were aged 60+ years and residents of Western Australia with a hospital record in the HMDS with any one of the following International Classification for Diseases (ICD-10-AM) codes for “dementia” as a principal diagnosis or a co-morbid condition from 2001 to 2013: Alzheimer’s dementia G30; vascular dementia F01; fronto-temporal dementia G31.0; Creutzfeldt-Jakob disease A81.0; dementia in Huntington’s disease F02.2; dementia in Parkinson’s disease F02.3; non-specific dementia F02.8, F03, F05.1, G31.1, G31.8, G31.9. Those with only drug, alcohol or HIV-related dementia were excluded. To ensure misclassification of exposure did not occur, each participant’s hospital records were searched from 1970 onwards to identify those with a previous hospital record that included dementia as a principal diagnosis or a co-morbid condition and were excluded from the analysis. The first admission with ‘dementia’ between 2001 and 2013 was considered the index admission. Because of the low diagnostic accuracy of specific dementia sub-diagnoses found in the HMDS, the analysis was undertaken for all cases of dementia [15].

### Study data

Once dementia cases were selected, de-identified injurious falls data including all hospital as well as death records from 2001 to 2013 were obtained using the WADLS. The hospital records include only those who were admitted to hospital for more than 24 h and do not include those who visited an Emergency Department only. Participants were defined as being hospitalized for an injury due to a fall if the primary diagnosis was an ‘*injury*’ as designated by a diagnosis code between S00.0 and T14.9 (Chapter XIX, ICD-10-AM), with a primary external cause code that identified a ‘*fall*’: W00-W19 (ICD-10-AM [16]; July 1, 1998–2008). If the main diagnosis was not an ‘*injury*’ but the participant fell then they were not included in the analysis. This was to ensure that the fall did indeed cause an injury and was not the result of another health related condition such as stroke.

Socio-demographic data was also extracted for all study participants which included age, gender, marital status, comorbidities, and residential location. Residential location was defined as metropolitan, rural or remote based on residential postcode at index admission. Marital status was classified as having a partner (married or de facto) or not (separated, divorced, or widowed). A comorbid health condition was classified as having one or more of the following 17 conditions described by Holman et al. [14] recorded during a hospital admission as a main diagnosis or a comorbidity, in the 5 year period prior to and including the index hospital admission. These comorbid conditions include myocardial infarction, diabetes, congestive heart failure, peripheral vascular disease, cerebrovascular disease, chronic pulmonary disease, rheumatic disease, peptic ulcer disease, hemi- or paraplegia, renal disease, tumors, lymphoma, leukemia, liver disease, metastatic solid tumor, and acquired immuno-deficiency syndrome. Dementia was excluded as it was the condition under study. Previous research examining lookback periods for co-morbid conditions found that 5 years was the most appropriate lookback period given that the effects of different comorbid conditions may vary depending on the conditions, their recency and duration [17]. An un-weighted co-morbidity score was assigned to each patient as the cumulative number of different co-morbid conditions identified. The presence or absence of co-morbid conditions was subsequently used in the analysis. A history of a hospitalization fall in the year prior to the index hospital admission with dementia was also included as this has been found to be a risk factor for multiple falls [7, 18].

### Statistical analysis

Descriptive statistics were used to summarize the characteristics of the cohort. Recurrent injurious falls in this study refer to more than one injurious fall. Thus the risk of recurrent injurious falls refers to the risk of subsequent injurious falls after a first fall has occurred.

Survival analysis using a stratified conditional Cox model (type 1) was undertaken after adjusting for relevant confounders which included age, gender, marital status, residential location, co-morbid conditions and an injurious fall in the previous year prior to the index hospital admission with dementia. The model included all those with dementia who had one or more injurious falls to determine risk factors for subsequent injurious falls after the first injurious fall had occurred. The stratified Cox model (type 1) method allowed an examination of the entire pattern of recurrent injurious falls and accounted for the highly correlated nature of the data. The stratified Cox model (type 1) also assumed that recurrent events were not identical and that each participant remained at risk for an injurious fall until the last interval was completed (last failure time or censorship).

Each participant’s survival time was calculated either from the first diagnosis of dementia to the first injurious fall and then the time between each subsequent injurious fall. Each new injurious fall was treated separately while assuming a common baseline hazard for each fall. The overall standard errors were adjusted for dependence which provided unbiased risk estimates [19]. The data for each patient was censored at either the date of death recorded in the WA Death Registrations or the end of the study period, 31st December 2013. Data analysis was undertaken using STATA (version 12) and results were considered significant at the 0.05 level.

## Results

There were 32,519 participants who had an index hospital admission for dementia from 2001 to 2013. The average age of the dementia cohort was 83.30 years (SD = 0.04). The majority were female (61.25 %), not married (56.73 %), lived in the metropolitan area (73.67 %) and had at least one medical comorbid condition (67.61 %). Nearly half of the cohort were aged 85+ years (47.95 %) and over a quarter (27.61 %) had been hospitalized due to an injurious fall in the year prior to the index hospital admission with dementia (Table 1). Eighty percent (*n* = 26,109) died during the study period.

**Table 1:** Characteristics of 32,519 Western Australian dementia patients by number of injurious falls observed, 2001–2013

Variable	N	%	No injurious falls observed (*n* = 23,549 participants)	%	1 injurious fall observed (*n* = 7297 participants)	%	2 or more injurious falls observed (*n* = 1673 participants)	%
Gender								
Female	19,919	61.25	13,309	56.52	5247	71.91	1363	81.47
Male	12,600	38.75	10,240	43.48	2050	28.09	310	18.53
Age group (years)								
60–64	122	0.35	112	0.48	10	0.14	0	0.00
65–69	1063	3.27	948	4.03	99	1.36	16	0.96
70–74	2357	7.24	1997	8.48	313	4.29	47	2.81
75–79	5159	15.86	4173	17.72	831	11.39	155	9.26
80–84	8225	25.29	6168	26.19	1712	23.46	345	20.68
≥ 85	15,593	47.95	10,151	43.11	4332	59.37	1110	66.29
Residential location^a^								
Metropolitan	23,938	73.67	17,197	73.08	5417	74.26	1219	72.88
Rural	5913	18.20	4347	18.47	1326	18.18	313	18.73
Remote	2642	8.13	1987	8.44	552	7.56	140	8.39
Co-morbidity								
No	10,532	32.39	6897	29.29	2864	39.25	771	46.08
Yes	21,987	67.61	16,652	70.71	4433	60.75	902	53.92
Marital status^a^								
Married	13,726	43.27	10,490	45.63	2526	35.29	476	28.60
^b^Not married	17,996	56.73	12,496	54.36	4631	63.46	1188	71.39
Inurious falls in previous year^c^								
No fall	23,540	72.39	17,646	74.93	4902	67.18	992	59.29
Fall	8979	27.61	5903	25.07	2395	32.82	681	40.71

^a^ *missing information*

^b^ *Single/widow/divorced*

^c^ *before index hospital admission with dementia*

Seventy-two percent (*n* = 23,549) of the cohort did not experience an injurious fall throughout the study period. Over 27 % (*n* = 8970) of the cohort experienced a total of 11,073 injurious falls that required hospitalization during follow up with 7297 individuals experiencing a single fall, 1330 individuals experiencing two falls and 343 individuals experiencing three or more falls during follow up (Table 1). The median follow-up time for each individual was 2.49 years with the total follow-up time being 83,145 years.

**Fig. 1:**
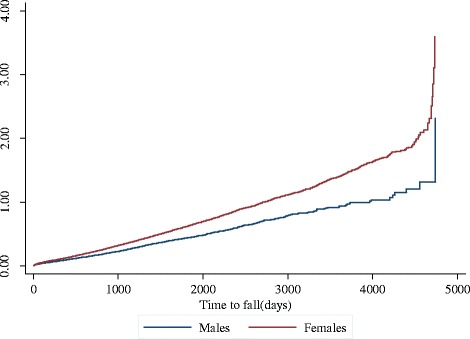
Risk of recurrent injurious falls for males and females

The results of the adjusted stratified conditional Cox survival analysis are shown in Table 2. Females were at a significantly increased risk of 7 % for recurrent injurious falls that required hospitalization (adjusted hazard ratio 1.07, 95 % CI 1.01–1.12) compared to males (Table 2 and Fig. 1). The risk of recurrent injurious falls also significantly increased with age. Compared to those aged 60 to 64 years, the risk of recurrent injurious falls was 2.11 times higher for those aged 70 to 74 years, 2.68 times higher for those aged 75 to 79 years, 3.68 times higher for those aged 80–84 and 5.78 times higher for those aged 85+. Living in the rural area significantly increased the risk of recurrent injurious falls resulting in hospitalization by 8 % (adjusted HR 1.08, 95 % CI 1.02–1.15) compared to those in the metropolitan area. Also, having an injurious fall in the year prior to the hospital admission with dementia significantly increased the risk of recurrent injurious falls by 8 % compared to those who did not have a fall (adjusted HR 1.08, 95 % CI 1.02–1.13).

**Table 2:** Hazard ratios using a stratified conditional Cox model for recurrent injurious falls risk

Variable	Adjusted hazard ratio	95 % confidence interval	*p*-value
Gender			
Male	1.00		
Female	1.07	1.01–1.12	0.009*
Age (years)			
60–64	1.00		
65–69	1.49	0.59–2.59	0.15
70–74	2.11	1.23–3.60	<0.001*
75–79	2.68	1.57–4.55	<0.001*
80–84	3.68	2.22–6.41	<0.001*
≥ 85	5.78	3.41–9.81	<0.001*
^a^Injurious fall in previous year			
No fall	1.00		
Fall	1.08	1.02–1.13	0.02*
Marital status			
Not Married	1.00		
Married	0.99	0.95–1.04	0.98
Comorbidity			
No	1.00		
Yes	0.97	0.92–1.01	0.25
Residential location			
Metropolitan	1.00		
Rural	1.08	1.02–1.15	0.008*
Remote	0.88	0.81–1.01	0.09

*Significant at p < 0.05

^a^ *Prior to index hospital admission*

## Discussion

In both the general population and those with dementia, the risk of falls is influenced by multiple factors which can include a previous history of a fall, impairment of balance, muscle strength, co-ordination and gait, impaired vision, functional impairment, medication usage, impaired cognition and mood, environmental hazards and inappropriate footwear [20]. In addition, for those with dementia, Harlein et al. found that the type and severity of dementia, behavioral disturbances, neuroleptic medication and low bone mineral density were risk factors for a fall [8]. In the present study, we used administrative health data sets to examine risk factors for recurrent injurious falls that required hospitalization for those with dementia at the population level, after adjusting for relevant confounders. The study found an increased risk of recurrent injurious falls for females, those aged 70 years and older, those living in rural areas and those who had experienced an injurious fall in the year prior to a hospital admission with dementia.

As expected, increasing age was a significant risk factor for recurrent injurious falls with an observed dose-response evident. As with the general population, the risk of falls increases with age for those with dementia and frailty associated with increasing age results in more severe injuries in the event of a fall. [21, 22]. Also consistent with previous research, a history of injurious falls in the year before hospital admission with a diagnosis of dementia was found to be associated with an increased risk of recurrent injurious falls in older adults with dementia. [7, 18] This finding suggests that it is essential to screen all those with dementia for injurious falls history in order to identify those most at risk of recurrent injurious falls.

This study found an increased risk of recurrent injurious falls resulting in hospitalization for females with dementia. Strong evidence suggests that among the general older population, females are at increased risk of falls [23]. However, conflicting evidence exists on the effect of gender on falls risk for those with dementia. Three studies of dementia patients in residential care facilities all reported that males were at increased risk of falls [13, 24, 25]. Another small study reported that males with Lewy body dementia and females with Alzheimer’s Disease experienced more multiple falls but the study only had a 3 month follow-up period [12]. It is known that females are more likely than males to sustain injuries such as fractures in the event of a fall [26]. Since our study specifically examined more severe falls resulting in hospitalization, this increased risk of injury for females may provide an explanation for the findings.

Those living in rural areas of WA were also at increased risk of recurrent injurious falls resulting in hospitalization. WA spans approximately two and a half million square kilometers is size, with around 70 % of the population living in metropolitan Perth. While health services are highly accessible in Perth, they are much less available in rural and remote WA [27]. Similarly, remoteness has been reported to be a risk factor for hospitalization and prolonged hospitalization among those with chronic diseases in WA [28]. More recently, studies have shown that remoteness was associated with increased duration of hospitalization in the last year of life for dementia patients in WA [29] and lower prescription rates of cholinesterase inhibitors for Alzheimer’s Disease in WA [30], highlighting possible inequities in levels of care for people with dementia in rural and remote areas. Therefore, our finding may reflect that those in rural areas have lower access to health care and falls prevention care both after diagnosis with dementia and after an initial fall resulting in hospitalization. Interestingly, our study did not find an increased risk of recurrent injurious falls resulting in hospitalization for those living in remote areas of WA. It is possible that as dementia progresses, or an individual experiences their first fall resulting in hospitalization, those living in remote areas have to move to a regional centre or the metropolitan area to receive the higher level of care they require. This warrants further investigation.

In this study, a large majority of the cohort with dementia (67.6 %) reported at least one comorbid health condition, however this did not significantly affect the risk of recurrent injurious falls. In fact, the proportion of those with (53 %) and without (46 %) a comorbid health condition who had two or more falls was not significantly different (*p* = 0.62). A possible reason for this is that the first fall may have resulted in a higher level of injury for those with a co-morbid condition, leading to lower mobility, reliance on aids such as wheelchairs or lower levels of activity, reducing the risk of recurrent falls. The fall may also have resulted in a higher level of care being arranged for those with a co-morbid condition, again reducing the risk of recurrent falls. It would be worthwhile to investigate this in a future longitudinal cohort study.

Unfortunately it was not possible to determine any concurrent use of medications that may have contributed to the fall as this information was not available in the WADLS. It is well documented that both older adults with and without dementia are commonly prescribed centrally acting medications such as antipsychotics, antidepressants and benzodiazepines, which can increase the risk of falls [31–33]. However, medications are modifiable risk factors and medication reviews have been shown to successfully reduce falls in the older population [34].

A major strength of this study was the use of statistical methods that modelled the recurrence of falls, as other methods such as logistic regression ignore repeated falls and relevant information is lost. The use of multifailure analysis allowed a more rigorous analytical approach to be used which accounted for correlation within individuals, longer follow-up time as well as time-varying covariates. Methods that model time-to-event data allow important risk factors to change over time and add to the robustness of the parameter estimates. The use of the WADLS has the advantage of reduced selection bias, minimal loss to follow-up, high quality, objective data and examination of the problem at the whole-population level [14]. A large majority of previous studies examining falls risk have been limited by small sample sizes of dementia patients [11, 35]. We were also able to adjust for comorbid health conditions such as diabetes and heart disease which are well-known risk factors for a fall [36, 37].

One limitation of the study is that dementia may not have been the primary diagnosis for the index hospital admission, however, it is often a co-morbid condition and may go unrecorded in the HMDS [38]. Therefore while the number of participants with dementia was possibly underestimated in our study, this underestimation would in fact result in our estimates being conservative and less than the true results. Another limitation of the study is that we were not able to determine the exact onset of dementia or the severity of the disease which would likely impact on recurrent injurious falls risk. While the index hospital admission was used as the best available estimate of dementia onset, it cannot be presumed that participants did not receive a dementia diagnosis prior to this. Several studies have shown that falls are most likely to occur in people with moderate severity of dementia who are still mobile but may need assistance when walking or rising from a chair [32, 39]. We also were unable to examine the association between specific types of dementia and risk of recurrent injurious falls resulting in hospitalization. Previous research has found differences in fall rates between those with different types of dementia. For example, a 6 month study of 110 older adults with dementia found that those with vascular dementia were twice as likely to fall as those with Alzheimer’s Disease [40]. However, due to the low diagnostic accuracy of specific dementia sub-diagnoses in the HMDS [41], the decision was made to analyze all cases of dementia.

The WADLS data also did not capture lifestyle factors, the type of residence the person was living in and physical activity levels, which may all influence falls risk. Information on the circumstance and location of the fall was also not available. It is well documented that older adults with dementia in aged care facilities and in hospitals are at a greater risk for a fall-related injury than those living in the community [35]. The addition of this information on the circumstance and location of the fall to the linked databases would provide valuable information that would help to determine the cause of the fall. It has been suggested that there are different casual pathways for falls in people with dementia compared to the general population [42] and a better understanding of these causes could be used to guide falls prevention initiatives. Finally, it should be noted that the data analyzed from the WADLS represented only the more severe cases of falls that required hospitalization for more than 24 h. It should also be acknowledged that having a diagnosis of dementia may have increased the need for hospitalization in the event of a fall due to social reasons, rather than the injury, especially if a person was living alone.

Despite these limitations, this study provides important evidence on risk factors for recurrent injurious falls resulting in hospitalization for older adults with dementia. It is known that those with dementia recover less well after a fall than those without the disease [43], meaning effective falls prevention services, targeted at those most at risk of injury are essential to reduce the burden of falls among older adults with dementia.

## Conclusions

In conclusion, this study used administrative health data sets to examine risk factors for recurrent injurious falls that required hospitalization for those with dementia at the population level. It found an increased risk of recurrent falls for females, those aged 70 years and older, those living in rural areas and those who had experienced a fall in the year prior to a hospital admission with dementia. The findings of this study suggest that screening for injurious falls history could help to identify those most at risk of injurious falls. In addition, improvement of heath care and falls prevention services for those with dementia who live in rural areas may also reduce the risk of injurious falls. While there are no proven interventions to prevent falls in people with dementia, promising interventions including safe exercise programs [44, 45] and home environment modifications [45]. Finally, given the increasing number of people who will be diagnosed with dementia as well as the fact that the mechanism for falls risk for older people with dementia is multifactorial, a large prospective cohort study is warranted to better clarify the determinants of risk which are not possible using the linked databases.

## Abbreviations

HMDS: Hospital Morbidity Data System
WA: Western Australia
WADLS: Western Australian Data Linkage System
